# Pediatric appendicitis score: A retrospective analysis

**DOI:** 10.4103/0971-9261.44761

**Published:** 2008

**Authors:** F. Goulder, T. Simpson

**Affiliations:** Department of General Surgery, Kent and Sussex Hospital, UK

**Keywords:** Appendicectomy, appendicitis, pediatric appendicitis score

## Abstract

**Aims:**

Evaluation of the pediatric appendicitis score (PAS), in all patients who had an appendicectomy over a one-year period.

**Methods:**

Retrospective study of 56 patients aged 4–15 years, who underwent an emergency appendicectomy. PAS was applied and patients were divided according to the PAS protocol into high probability and low probability groups. These results were then correlated with histology.

**Results:**

The PAS had sensitivity 0.87, specificity 0.59, positive predictive value 0.83, and negative predictive value 0.67. The negative appendicectomy rate would have been reduced to 17%, but five patients with appendicitis would have been denied early surgical treatment and may have been discharged.

**Conclusions:**

The PAS cannot be recommended as it would lead to an unacceptable risk of wrongly discharging or delaying necessary surgery in 13% of patients with appendicitis.

## INTRODUCTION

Acute appendicitis is one of the most frequent general surgical emergencies, accounting for over 40,000 hospital admissions in England every year.[1] In many cases a careful history, examination, and simple bedside tests are all that are required to make the diagnosis.[1] Risk of perforation increases significantly 24 hours following admission.[2]

The investigation of patients with possible appendicitis varies widely between hospitals and countries, and there are many conflicting recommendations within the international literature (blood counts, ultrasonography, CT/MRI scans).[3–10]

Studies have also shown up to 46% of appendicectomies being performed in patients, when not required (normal appendix at histological examination). Though various scoring systems have been introduced, none so far has been independently demonstrated to fulfill even the basic requirements regarding sensitivity and specificity although the authors responsible for introducing each method have generally produced good results in their own units.[11]

The aim of this study was to analyze the accuracy of the pediatric appendicitis score (PAS)[12] by retrospectively scoring pediatric patients who have undergone an appendicectomy and comparing the patients’ scores with their appendix histology.

## PATIENTS AND METHODS

Hospital records of all patients aged 4–15 years who underwent an emergency appendicectomy as a sole procedure at the Kent and Sussex Hospital over a one-year period were retrieved. Hospital records identified 69 patients. Out of 60 available charts, 56 of these contained sufficient information to accurately apply the scoring system [Table 1]. After data analysis, the patients were placed into one of two groups: Either PAS ≤ 5 or PAS ≥ 6. The histology was not reviewed until after the final result had been recorded. The PAS for each patient was then compared with the true histology results.

**Table 1:** Pediatric appendicitis score[12]

Symptom	Score
Anorexia	1
Pyrexia	1
Nausea or vomiting	1
Migration of pain	1
Raised white cell count	1
Raised neutrophil count	1
RIF tenderness	2
Cough/percussion/hopping tenderness	2

## RESULTS

The median age of these patients was 12 years (range, 7–15) with an equal male:female ratio (28 male and 28 female patients).

The PAS was applied to each patient's notes and they were classified into four groups according to PAS and histology: true positive score (PAS ≥ 6, histology positive), true negative score (PAS ≤ 5, histology negative), false positive score (PAS ≥ 6, histology negative), and false negative score (PAS ≤ 5, histology positive) as shown in Table 2.

**Table 2:** Pediatric appendicitis score compared to histology

	Histology = normal	Histology = appendicitis
PAS = 1−5	10 patients	5 patients
PAS = 6−10	7 patients	34 patients

The PAS has a sensitivity of 0.87, specificity of 0.59, positive predictive value of 0.83, and negative predictive value of 0.67. Had we used the PAS in the diagnosis of these 56 patients, our negative appendicectomy rate would have been reduced to 17%. However, five patients with appendicitis would have been discharged or had their treatment delayed by a false negative score.

## DISCUSSION

The PAS is a relatively new scoring system which relies upon simple points in the history and examination of a patient. It is scored out of ten with a score of five or less excluding appendicitis, and a score of six or above making a true case of appendicitis highly likely.[12] The author of the study introducing this scoring system reported a sensitivity of 1, specificity of 0.92, positive predictive value 0.96, and negative predictive value of 0.99. This would result in a 4% negative appendicectomy rate, and no patients with appendicitis being discharged or receiving delayed treatment as every case of appendicitis would be diagnosed accurately.

The negative appendicectomy rate in the pediatric population is significantly higher than that in any other age group, and can be up to 46%. The PAS is a diagnostic tool which is simple to apply and requires no additional resources. According to our data this scoring system has a positive predictive value of 0.83 and a negative predictive value of 0.67.

From these results, if the PAS were introduced and used as the sole factor in deciding whether or not to operate in cases of suspected appendicitis, the negative appendicectomy rate would be 17%. More worryingly, however, using this scoring system would result in 13% of all cases of appendicitis being missed and potentially discharged.

These results are comparable with those published by Lintula *et al*, who recently introduced an alternative PAS system.[13] Their diagnostic score incorporates gender, intensity of pain, relocation of pain, vomiting, right lower quadrant pain, abnormal bowel sounds, fever, guarding, and rebound tenderness to create a score out of 32. Using this scoring system, three groups are identified: score ≥ 21 indicates a high probability of appendicitis (appendicectomy required); score ≤ 15 indicates a low probability of appendicitis (patient can be discharged); score = 16−20 indicates an intermediate probability of appendicitis (patient should be observed). Despite introducing the ‘safety net’ of an intermediate group for ongoing clinical assessment, this scoring system is said to result in 15% of patients with appendicitis receiving a score of ≤15 and therefore being discharged. In addition, if all the patients scoring ≥21 were operated on, the negative appendicectomy rate would be 13%. These figures do not take into account the management of those patients in the intermediate/observation group, often the most difficult to manage. The negative appendicectomy rate would increase if this group were included in the analysis. The authors concluded that while their scoring system can be used as a diagnostic aid, it cannot supplant careful clinical judgment.[13]

With reference to the PAS assessed in this study, we have not been able to reproduce the impressive results reported earlier.[12] Rather, we have found the score to result in a negative appendicectomy rate of 17%, and an unacceptable 13% of patients with appendicitis being missed. Given the significant increase in morbidity and mortality associated with a delay in the diagnosis of appendicitis, the PAS cannot be recommended from our data.
